# Characterization and Comparison of Structure and Physicochemical Properties of Highland Barley Starch of Different Colors

**DOI:** 10.3390/foods14020186

**Published:** 2025-01-09

**Authors:** Mengru Han, Xiongying Zhang, Honglu Wang, Jiayue Zhou, Meijin Liu, Xirong Zhou, Aliaksandr Ivanistau, Qinghua Yang, Baili Feng

**Affiliations:** 1 College of Agronomy, Northwest A&F University, Yangling 712000, China; hanmengru@nwsuaf.edu.cn (M.H.); xn20210909@163.com (X.Z.); 2018050099@nwsuaf.edu.cn (H.W.); yahaha@nwafu.edu.cn (J.Z.); qinghuayang@nwsuaf.edu.cn (Q.Y.); 2 Gannan Tibetan Autonomous Prefecture Agricultural Science Research Institute, Hezuo 747000, China; llmmjj8989@163.com (M.L.); 13519316009@163.com (X.Z.); 3 Belarusian State Agricultural Academy, 213407 Gorki, Belarus; ivanistov09@mail.ru

**Keywords:** highland barley, grain color, starch, structural properties

## Abstract

Domesticated highland barley is an important starch reserve and has differently colored grains, owing to different genotype backgrounds and cultivation environments. In this study, black, purple, blue, and yellow highland barley varieties were planted under the same cultivation conditions, and their starch distribution, structural characteristics, and physicochemical properties were analyzed. The apparent amylose content was highest in the purple variety (20.26%) and lowest in the yellow variety (18.58%). The different varieties had three subgroups and A-type crystalline structures, but the particle size and relative crystallinity (25.67–27.59%) were significantly different. In addition, the weight average molecular weight (6.72 × 10^7^ g/mol), area ratio of APs to AP_L_ (2.88), relative crystallinity (27.59%), and 1045/1022 (0.730 cm^−1^) of starch were higher in yellow highland barley (YHB), forming a stable particle structure and increasing the Tp and PV of its starch. A cluster heat map showed that starches from differently colored highland barley vary in fine structure, water solubility, swelling power, and thermal and pasting properties. This study provides a reference for the high-quality breeding of colored highland barley and its utilization in food and non-food industries.

## 1. Introduction

Highland barley (*Hordeum vulgare* var. *coeleste* Linnaeus) is one of the varieties of barley and the main crop in the Qinghai-Tibet Plateau, which includes Qinghai and Tibet in China, Nepal, and Bhutan [1]. Its grain is rich in starch, vitamins, dietary fiber, and minerals, which are sources of dietary energy for people in high-altitude areas, and is an important source of starch in food (noodles, beer) and non-food industries (animal feed). In recent years, the requirements for dietary quality have greatly improved; therefore, multicolor cereals with outstanding antioxidant capacities have attracted much attention. Among them, the color of highland barley grains is diverse (e.g., black, blue, and yellow/white) owing to the influence of grain pigment content and phenolic compounds, and differences in grain color lead to changes in nutritional and functional components [2].

Starch accounts for 47.9–79.0% of highland barley grains, serving as their main component, and is higher in highland barley than in hulled barley [3]. Starch includes amylose (linear, low molecular weight) and amylopectin (highly branched, high molecular weight) [4]. The amylopectin content of highland barley is >70%. The prominence of starch has aroused interest in its structure and properties, as well as its quality and application in the (non-) food industry. The functional properties of highland barley have been widely studied in different grain colors [1,2,3,5], but there are few reports on the structure and properties of colored highland barley starch. The properties and functionalities of starch vary among different varieties of the same plant species [6]. Guo et al. (2019) [7] and Soison et al. (2015) [8] used colored sweet potato starch as a research material, and found that different genotypes determined its physicochemical properties. Similarly, Sompong et al. (2011) [9] reported amylose contents ranging from 7.4% to 42.0% in ten red-pericarp rice varieties and from 8.9% to 25.4% in three black-pericarp varieties, which were attributed to genetic, environmental, and cultivation factors. Ziegler et al. (2017) [10] found that starch isolated from rice grains with brown, black, and red pericarps displayed varying amylose contents after storage at different temperatures, which were mainly influenced by genetic and environmental conditions, rather than pericarp color. Trehan et al. (2020) [11] investigated the grain, flour, and starch properties of 38 colored maize types. Despite the focus on the starch properties of other colored crops, colored highland barley remains an underexplored but valuable germplasm resource. The structure and physicochemical properties of starch are important for highland barley quality in the breeding and application of highland barley.

The differences in the starch properties of highland barley varieties of different colors are unclear owing to the diversity of their genotypic backgrounds. It is necessary to characterize and comprehensively compare the structural and physicochemical properties of highland barley starch from the perspective of different colors. In this study, the starch structure and physicochemical properties of black, purple, blue, and yellow highland barley were compared and analyzed. Finally, the correlations between the starch properties of highland barley of different colors were discussed using cluster heat map analysis. In this study, the starch properties of colored highland barley were characterized and comprehensively compared to provide a useful reference for the high-quality breeding and industrial utilization of highland barley varieties of different colors.

## 2. Materials and Methods

### 2.1. Materials

**Figure 1:**
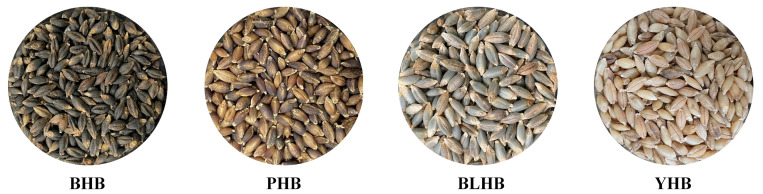
The four highland barley varieties of different colors. Note: BHB, PHB, BLHB, and YHB represent black highland barley, purple highland barley, blue highland barley, and yellow highland barley, respectively.

A black variety (BHB, Liuleng), purple variety (PHB, Gannan0835), blue variety (BLHB, Gannan0917), and yellow variety (YHB, Ganqing10) of highland barley were selected as the experimental materials. The chemical constituents of the highland barley varieties are shown in the Supplementary Materials (Table S1). The BHB, PHB, BLHB, and YHB were planted in the same experimental plot at the Gannan Tibetan Autonomous Prefecture Agricultural Science Research Institute (N35°0′, E102°54′, approximately 2960 m altitude), and were used after harvest in 2022 (Figure 1). The samples were dried at room temperature (20 ± 5 °C) after harvest. The colored highland barley grains were pulverized with a high-speed crusher, and then the whole flours were sieved through a 100-mesh sieve and stored at 4 °C for future use.

### 2.2. Starch Preparation

Starch was isolated from the four highland barley flours according to the method described by Obadi et al. (2021) [3]. The flour of the highland barley was soaked in 0.3% NaOH solution at a ratio of 1:5 (g/mL) and extracted for 12 h at 25 °C. The samples were passed through 100-mesh and 200-mesh sieves, then centrifuged (5000× *g* for 10 min). The supernatant was removed, and the wet starch from the bottom was washed. Finally, the wet starch was dried at 40 °C, ground, and sifted (100-mesh sieve).

### 2.3. Scanning Electron Microscopy

The morphologies of the starch granule surfaces were observed using scanning electron microscopy (SEM, Nano SEM-450, FEI, Hillsboro, OR, USA), following previously reported methods [12]. Micrographs of the starch were obtained at 1500× and 6000× magnification. Starch granule size was determined by measuring 50 granules with complete morphology using ImageJ 1.54g software, and a frequency distribution histogram was drawn for the size results.

### 2.4. Granule Size

The starch size of highland barley was measured using a laser diffraction particle size analyzer (Mastersizer 2000, Malvern Instruments, Malvern, UK) [13].

### 2.5. Granule Distribution

The size and complexity of the highland barley starch were analyzed by flow cytometry (BD FACSAriaIII, Franklin Lakes, NJ, USA) based on the method described by Zhang et al. (2018) [14], with some modifications. Unstained starch granules were used as the negative controls.

### 2.6. Apparent Amylose Content (AAC) Analysis

AAC was measured using the iodine colorimetric method described by Gao et al. (2022) [15]. Briefly, 0.01 g of starch successively received 100 μL of anhydrous ethanol and NaOH (1 mL, 1 mol/L) solution, then 8.9 mL distilled water was added to prepare the stock solution. Next, 200 μL stock solution was mixed with hydrochloric acid (200 μL, 0.1 mol/L) and iodine solution (200 μL). The absorbance of the samples was measured at 620 and 510 nm.

### 2.7. Structural Characteristics of Starch

#### 2.7.1. Molecular Weight Distribution

Starch (5 mg) was mixed with 5 mL of DMSO solution to prepare a suspension. The average molecular weight and polydispersity index of each component of the sample were measured using a laser photometer (DAWN HELEOS-II, Wyatt Technology Co., Goleta, CA, USA).

#### 2.7.2. Chain Length Distribution

Starch (5 mg) and distilled water (0.9 mL) were mixed in centrifuge tubes and boiled in boiling water for 15 min. Next, 5 mL of 40 mg/mL sodium azide solution, 0.1 mL of acetate buffer, and 10 uL of isoamylase (1400 U) were added to mixture. Debranched starch was precipitated in 5 mL anhydrous ethanol and centrifuged (4000× *g* for 10 min). The solution was then oscillated with MSO/LiBr solution (80 °C, 2 h). Finally, a differential refractive index detector (Optilab T-rEX, Wyatt Technology Co., Santa Barbara, CA, USA) was used to determine the molecular weight distribution of the debranched starches.

#### 2.7.3. X-Ray Diffraction

The crystalline structure of the starch was determined by XRD (D/Max 2550 VB +/PC, Rigaku Corporation, Tokyo, Japan) using the method described by Yangcheng et al. (2016) [12], with slight modifications. The test parameters of the diffractometer were as follows: the voltage was 40 kV and 100 mA, and the scattering angle range was 5–50°.

#### 2.7.4. Fourier Transform Infrared Spectroscopy (FTIR)

The ordered starch structures were analyzed by FTIR (Nicolet iS10, Thermo Fisher Scientific, Waltham, MA, USA). Starch, evenly spread on the ATR mold surface, was penetrated by an infrared beam. The test parameters were as follows: FTIR spectrum was 1200–800 cm^−1^, scanning time was 32, and resolution was 4 cm^−1^. Deconvolution of data was performed using OMNIC 8.2.

### 2.8. Physicochemical Properties of Starch

#### 2.8.1. Water Solubility (WS) and Swelling Power (SP)

The WS and SP of highland barley starch were analyzed at 75, 85, and 95 °C following a previously described method [16].

#### 2.8.2. Light Transmittance (LT)

A total of 0.2 g of highland barley starch and 20 mL of distilled water were mixed and gelatinized for 30 min. Then, the gelatinized sample was cooled to room temperature. The LT was measured at 620 nm, and distilled water was used as the control [17].

#### 2.8.3. Thermal Properties

The thermal properties of the highland barley starch were analyzed using a differential scanning calorimeter (TA Q2000, Perkin Elmer Instruments, Waltham, MA, USA). Briefly, starch (3 g) and distilled water (9 µL) were placed in an aluminum pan and kept at 4 °C overnight. The sample was scanned from 40 to 100 °C and the scanning rate was 10 °C/min, and an empty pan was used for reference. The data were acquired using Universal Analysis 2000 [16].

#### 2.8.4. Pasting Properties

The pasting properties of the highland barley starch were measured using a Rapid Visco Analyzer (RVA 4500, Perten, Stockholm, Sweden) according to the method described by He and Wei (2017) [18], with slight modifications. The starch–water suspension (14% moisture, 28.0 g total mass) was held (50 °C, 1 min) and heated to 95 °C, and then held (95 °C, 2 min). Finally, the suspension was cooled to 50 °C at the same rate and allowed to rest. The pasting parameters of the starch samples were recorded.

### 2.9. Statistical Analysis

Data are expressed as mean ± standard deviation. Analysis of variance (ANOVA) and Duncan’s multiple range tests were performed using SPSS 17.0 statistical software. Cluster heatmap analyses and figures were plotted using Origin 2023 and Visio 2021, respectively.

## 3. Results and Discussion

### 3.1. Starch Morphological Properties

**Figure 2:**
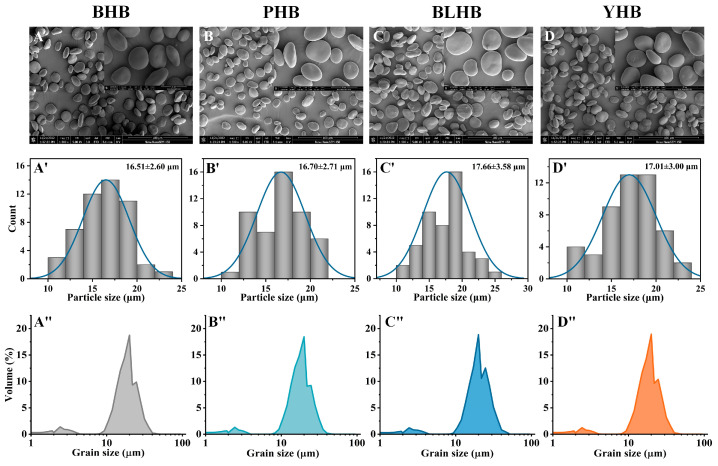
SEM images (magnification of 1500 and 6000) (**A**–**D**), relative image analysis (**A’**–**D’**), and granule sizes (**A”**–**D”**) of highland barley starch from four cultivars. Note: BHB, PHB, BLHB, and YHB represent black highland barley, purple highland barley, blue highland barley, and yellow highland barley, respectively.

The morphology of the highland barley starch granules was observed using SEM at magnifications of 1500× and 6000× (Figure 2A–D). The sample granule surfaces were smooth, with oval, elliptical, and disk shapes, and a small number of granules had spherical shapes. Chen et al. (2023) [2] and Li et al. (2014) [19] reported similar observations for different varieties of highland barley and naked barley starches. The starch source [20] and growth environment [21] are the main factors affecting the morphological characteristics of starch. Image analysis (Figure 2A’–D’) showed that the particle sizes of highland barley starch from varieties of different colors obeyed a normal distribution, and the granule sizes of different varieties were significantly different. Among them, the granule sizes of BLHB and BHB had the maximum value (17.66 μm) and the minimum value (16.51 μm), respectively.

The granule distribution (volume distribution and standard average diameter) of the highland barley starch was analyzed. The volume distribution of the highland barley starch showed bimodal distribution with small granules (about 2.4 μm) and large granules (about 20 μm) (Figure 2A”–D”). The granule sizes of most of the highland barley starch ranged between 7 μm and 50 μm, which is smaller than that of potato starch [22] and larger than that of broomcorn millet and buckwheat starch [13,23]. The d (0.5) ranged from 16.63 to 18.65 μm, and the D [3, 2] and D [4,3] were in the range of 9.05 to 9.89 μm and 16.64 to 18.66 μm (Table 1), respectively, with BLHB (blue highland barley) having the maximum value and PHB (purple highland barley) having the minimum value. Among them, the BHB (black highland barley) and PHB starch granules were similar in size. The particle size of starch is affected by the plant variety, growth conditions [24], and plant physiology [23]. In this study, the four highland barley varieties were cultivated in the same experimental field, indicating that different genotype backgrounds cause differences in the particle size distribution of highland barley starch.

### 3.2. Granule Size Distribution

**Figure 3:**
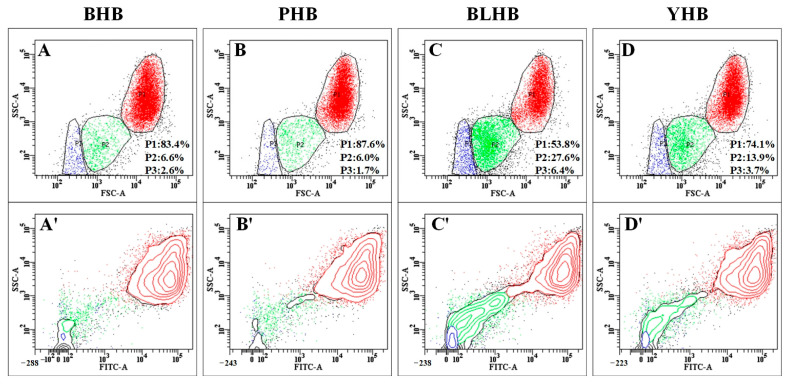
Bivariate flow cytometric histograms of highland barley starch from four cultivars. (**A**–**D**) forward scattered–side scattered (FSC-SSC) images; (**A’**–**D’**) fluorescence images. Note: BHB, PHB, BLHB, and YHB represent black highland barley, purple highland barley, blue highland barley, and yellow highland barley, respectively.

Further fractionation and analysis of the starch granules were performed using flow cytometry, to understand their size and complexity. This method is commonly used in the fractionation of crops such as corn [14], potatoes [6], and buckwheat [20]. Plots of FSC versus SSC and FITC/APTS versus SSC (Figure 3), which represent the structural complexity, size, and fluorescence intensity of the starch granules, respectively, were analyzed. The authenticity of these results was verified using unstained starch granules (Figure S1). As shown in Figure 3A–D, the highland barley starch was divided into three subgroups (P1, P2, and P3). The *p*-value of the same subgroup was significantly different among the varieties, with the largest P1 occurring in PHB, indicating that its particle size and structure are larger and more complex, respectively. In contrast, the P1 of BLHB was the smallest, indicating that there are more small granules in BLHB than in the other varieties of highland barley. From the APTS-SSC plot (Figure 3A’–D’), most particles in the three subgroups were dyed. Yang et al. (2019) [20] divided the starch granules of buckwheat and sorghum into three subgroups, similar to our results.

### 3.3. Apparent Amylose Content (AAC)

AAC is an intuitive and useful structural parameter that affects starch properties. For example, amylose is closely related to starch pasting, retrogradation, and gelatinization properties [23,25]. Differences were observed in AAC, which ranged from 18.58 (BLHB) to 20.26% (PHB) (Table 1). The AAC values were lower than those of highland barley (approximately 23%) reported by Waleed et al. (2021) [26], and the difference was mainly affected by the determination method, crop variety, and growth environment [22]. In addition, Figure S2B shows that AAC is independent of highland barley grain color, which is consistent with the results of Sompong et al. (2011) [9] and Ziegler et al. (2017) [10] regarding colored rice and those of Guo et al. (2019) [24] for three-colored sweet potatoes. Different amylose concentrations are primarily a result of genetic factors, environmental conditions, and plant cultivation practices.

### 3.4. Analysis of Structural Characteristics

#### 3.4.1. Molecular Weight Distribution

**Figure 4:**
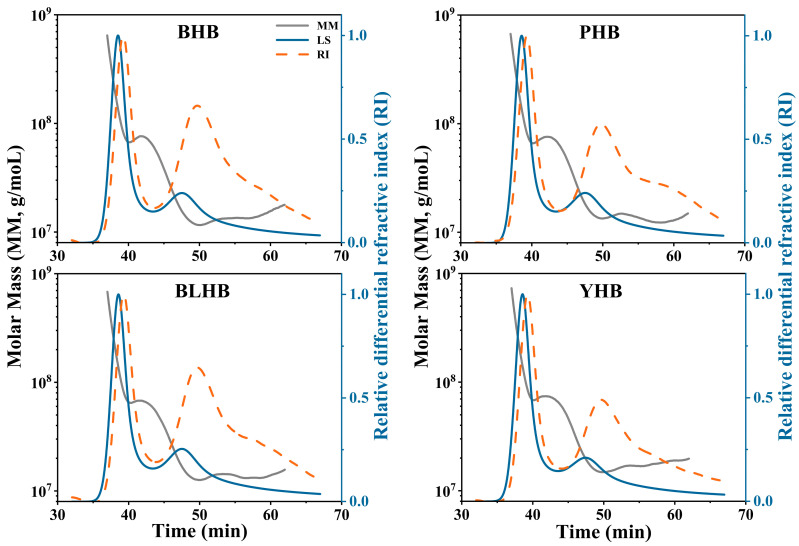
The molecular weight distribution of highland barley starch. Note: MM, molar mass; LS, light scattering signal; RI, refractive index. Note: BHB, PHB, BLHB, and YHB represent black highland barley, purple highland barley, blue highland barley, and yellow highland barley, respectively.

Differences in the degree of molecular weight distribution in highland barley starch from different color varieties are presented in Table 2 and Figure 4. The highland barley samples showed Mw (weight average molecular weight) of 5.43–6.72 × 10^7^ g/mol, Mn (number average molecular weight) of 1.99–2.68 × 10^7^ g/mol, and Mp (peak molecular weight) of 10.78–11.54 × 10^7^ g/mol. The Mw, Mn, and Mp values of the four samples were significantly different, and the molecular weight of yellow highland barley (YHB) was higher than that of the other varieties, which may be due to its high amylopectin content. Huang et al. (2015) [27] reported that the molecular weight of amylopectin is typically higher than that of amylose. You et al. (2015) [28] and Gao et al. (2020) [17] found that Mw in normal rice and buckwheat starch is 20.17 × 10^7^ g/mol and 4.30 × 10^7^ g/mol, respectively, while the average Mw of this study was 5.86 × 10^7^ g/mol. These differences are related to the genotypes and growth environments of the crop varieties. Lee et al. (2017) [29] reported that differences in relative molecular weight affect the physicochemical properties of rice starch. Therefore, molecular weight differences in highland barley may influence its physicochemical properties. In addition, black highland barley (BHB) starch had a lower Mw and radius of gyration (Rz), as well as a higher polydispersity index (PDI), and its starch digestibility and crystal thickness may be higher than those of PHB, BLHB, and YHB. The PDI of YHB was the lowest compared with that of the other highland barleys, indicating that the molecular weight distribution in YHB is narrower and its distribution curve is more uniform.

#### 3.4.2. Starch Chain Length Distribution

**Figure 5:**
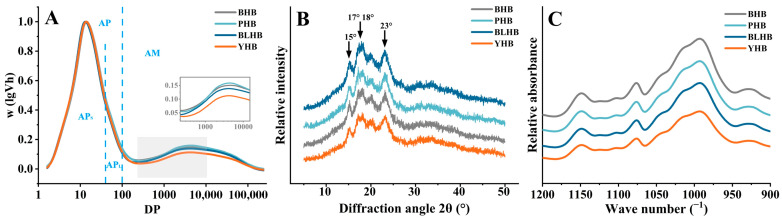
(**A**) chain length distribution, (**B**) XRD pattern, and (**C**) FITR spectrum of highland barley starch from four cultivars. Note: BHB, PHB, BLHB, and YHB represent black highland barley, purple highland barley, blue highland barley, and yellow highland barley, respectively. APs, amylopectin short-branch chains; AP_L_, amylopectin long-branch chains; AM, amylose chains; RSL, area ratio of APs to AP_L_.

The starch chain length distribution was characterized by gel permeation chromatography (GPC). Two obvious peaks were observed in all the curves (Figure 5A), and the GPC curves were divided into APs, AP_L_, and AM. The amylopectin (AP) and amylose (AM) were divided using DP100. Klucinec and Thompson (1999) [30] and Jane and Chen (1992) [31] suggest that the interaction between amylose-amylopectin and amylopectin-amylopectin is the reason for the change in gel properties during long-term storage. The APs of yellow highland barley (YHB, 61.62%) starch were higher than those of the other varieties, and their AP_L_ values were significantly different (Table 2). The AM of the highland barley starch was 16.93% to 22.18%. The AM value of PHB was significantly higher than that of the other highland barley varieties, which was attributed to their genotypes. The greater the value of the RSL (area ratio of APs to AP_L_), the greater the degree of branching in amylopectin. Thus, the degree of branching in YHB starch was significantly higher than that in the other starches.

#### 3.4.3. Starch Crystalline and Ordered Structure

The arrangement and composition of amylose and amylopectin determine the helical structure of starch crystals [32]. XRD has been widely used to investigate crystal structures on a larger scale [13]. The XRD patterns of highland barley starch are shown in Figure 5B. Starch crystals are classified into four types [18]: A-, B-, C-, and V-types. All samples were typical A-type starches based on the diffraction peak position, which is consistent with the diffraction peaks of highland barley starch reported by Chen et al. (2023) [2]. The XRD peaks of the highland barley starch were slightly different in this study, which may be due to the different highland barley varieties. The relative crystallinity ranged from 25.67% (PHB) to 27.59% (YHB) (Table 2), indicating that YHB has more crystalline regions than the other highland barley varieties. Khatun et al. (2019) [33] reported that the high crystallinity of starch improves its stability and reduces the probability of starch gelatinization and digestion. The relative crystallinity values of the four highland barley starches were slightly lower than those reported in previous studies [2], which was due to the genotypes and determination methods. The chain length and amylopectin content affect the relative crystallinity of starch, whereas amylose molecules are related to the amorphous region.

The short-range-ordered structures of the highland barley starch were analyzed using FTIR. All starches exhibited similar FTIR spectra (Figure 5C). The absorbance ratio can express the order degree (1045/1022) and amorphous (1022/995) ratios of the carbohydrate structures [34]. There were differences in the absorbance ratios of the four highland barley starches, but the 1045/1022 and 1022/995 values of YHB, which were 0.730 cm^−1^ and 0.834 cm^−1^, respectively, were significantly higher than those of the other varieties (Table 2). Chen et al. (2023) [2] found that the average value of 1045/1022 in blue highland barley is significantly higher than that in white highland barley, which is different from the results of this study, indicating that the variety affects the ordered structure of starch.

### 3.5. Analysis of Physicochemical Properties

#### 3.5.1. Starch Water Solubility (WS) and Swelling Power (SP)

**Figure 6:**
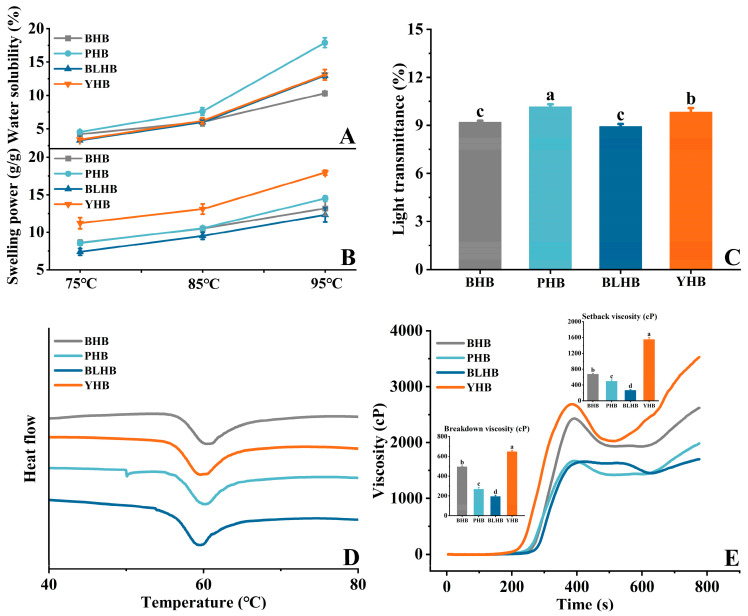
Physicochemical properties of highland barley starch from four cultivars. (**A**) Water solubility, (**B**) swelling power, (**C**) light transmittance, (**D**) thermal, (**E**) and pasting properties. Different letters indicate significant differences, *p* < 0.05. Note: BHB, PHB, BLHB, and YHB represent black highland barley, purple highland barley, blue highland barley, and yellow highland barley, respectively.

WS and SP reflect the interaction between starch and water, representing the degree of starch dissolution at different temperatures and pressures and starch water holding capacity, respectively [3]. As shown in Figure 6A,B and Table S2, the WS and SP of the highland barley starch changed markedly at different temperatures. As the temperature increased, the WS and SP values increased significantly. The WS and SP at 75 °C were lower than those at other temperatures, with average values of 3.83% and 8.95 g/g, respectively. At 95 °C, the WS and SP of all the samples reached their maximum values. This phenomenon occurred because the heating temperature of the starch was close to the gelatinization temperature, which gradually loosened its crystal structure. Subsequently, the starch granules absorbed the surrounding water owing to the combination of the starch polar groups with water, thereby increasing the WS and SP of the starch [19]. At the same temperature, PHB and YHB exhibited the maximum WS and SP, respectively. Obadi et al. (2021) [3] showed that the WS and SP of highland barley starch ranges from 13 to 19% and 12 to 17% at 90 °C, respectively. In general, WS and SP are often quite different at the same temperature, according to various studies. The WS and SP of starch are affected by the amylose-amylopectin ratio, molecular weight, chain length distribution, and morphological characteristics of the starch, such as granule size [35].

#### 3.5.2. Starch Light Transmittance (LT)

LT can reflect the bonding strength of starch and water, and the greater the paste LT, the better the transparency [13]. Figure 6C shows that the LT values of the four highland barley starches were different, ranging from 8.93% to 10.17%, with the highest value occurring in purple highland barley (PHB) and the lowest value in blue highland barley (BLHB), which indicates that more particles of PHB starch dispersed into the water and had the highest transmittance. The LT values in this study were significantly lower than those in previous studies [36], and this difference may be related to the varieties and the determination methods (such as a gelatinization time of 20 min or 30 min). In addition, Obadi et al. (2021) [3] showed that the LT of starch is also related to starch water solubility; the higher the water solubility, the better the light transmittance. Lu et al. (2016) [36] found that the LT of barley starch was higher than that of buckwheat and wheat starches. The changes in LT in the four different highland barley starches could be explained by differences in the starches’ water solubility, molecular structure arrangements, and amylose-amylopectin ratios [37].

#### 3.5.3. Starch Thermal Properties

Thermographs of the four highland barley starches are shown in Figure 6D, and the parameters of the different highland barley starches are summarized in Table S3. The thermogram curves of all samples were smooth overall. The gelatinization peak temperature (Tp) was the lowest in PHB and the highest in YHB, equaling 59.33 °C and 60.19 °C, respectively. Similar Tp results have been reported for naked barley starch [19]. Starch requires a higher temperature of dissolution owing to its higher gelatinization transition temperature [38]. The results show that purple highland barley (PHB) was the easiest of the four varieties to change from a crystalline to gel state through heating. Jie, Zhang, and Zhang (2010) [39] reported that gelatinization temperature of highland barley samples is lower than that of wheat starch, which indicates that highland barley starch is easier to gelatinize than wheat starch. This study shows that range of gelatinization enthalpy (ΔH) was 9.43–10.78 J/g, and the ΔH of BHB and PHB was lower than that of BLHB and YHB, suggesting that blue highland barley (BLHB) and yellow highland barley (YHB) have a more stable crystal structure, which is basically consistent with the results of relative crystallinity and granule size distribution reported above. The ΔH value of this study was close to the value of the highland barley starch studied by Obadi et al. (2021) [3]. Changes in gelatinization temperature and enthalpy are influenced by starch granule size, molecular structure, and main chemical components [40].

#### 3.5.4. Starch Pasting Properties

Starch’s pasting properties determine its quality and utilization [41]. The viscosity curve and pasting parameters of highland barley starch are shown in Figure 6E and Figure S3, respectively. The peak viscosity (PV) ranged from 1642 to 2660 cP, with the maximum and minimum values occurring in YHB and BLHB, respectively. The PV of starch represents the maximum expansion of its granules before disintegration and indicates the equilibrium point between granule expansion and breakdown [19]. The trough viscosity (TV) of PHB and YHB was in the range of 1445–2012 cP, corresponding to PHB and YHB, respectively. Breakdown viscosity (BV) is the difference between PV and TV, which are the destruction of expanded granules and the leaching of amylose into solution [40]. In this study, the final viscosity (FV) of the four samples was between 1716 (BLHB) and 3562 (YHB) cP, which was lower than the mean FV value of naked barley starch reported by Li et al. (2014) [19]. This difference may be related to the genotype and the interaction between starch components. The setback viscosity (SV) value was 268–1550 cP. SV indicates the retrogradation trend of starch after gelatinization [40], which indicates a lower retrogradation tendency for BLHB starch, which displayed the minimum SV. YHB showed the lowest pasting temperature (PT, 82.38 °C), while BLHB showed the highest (89.33 °C). Li et al. (2014) [19] reported that the high gelatinization temperature of starch indicates its high swelling resistance. In summary, the pasting properties of starch are significantly different among highland barley varieties of different colors.

### 3.6. Starch Cluster Heat Map Analysis

**Figure 7:**
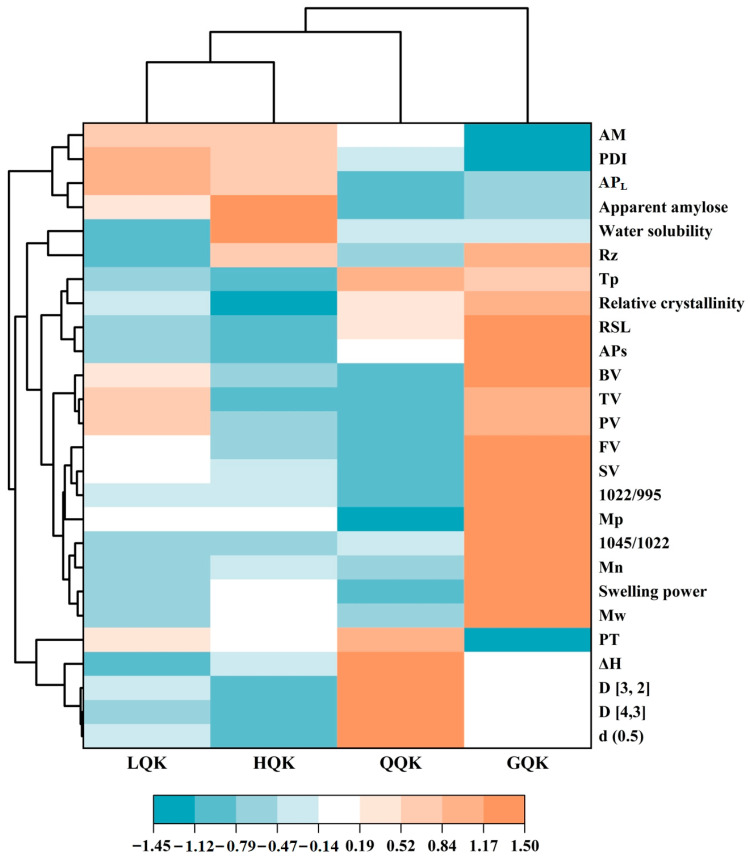
Cluster analysis based on starch property parameters from different colored highland barley varieties. Note: BHB, PHB, BLHB, and YHB represent black highland barley, purple highland barley, blue highland barley, and yellow highland barley, respectively. d (0.5), diameter corresponding to 50% of cumulative distribution of particle size; D [3, 2], surface-weighted average diameters of particles; D [4,3], volume-weighted average diameters of particles; Mw, weight average molecular weight; Mn, number average molecular weight; Mp, peak molecular weight; PDI, polydispersity index; Rz, radius of gyration; APs, amylopectin short-branch chains; AP_L_, amylopectin long-branch chains; AM, amylose chains; RSL, area ratio of APs to AP_L_; Tp, gelatinization peak temperature; ΔH, gelatinization enthalpy; PV, peak viscosity; TV, trough viscosity; BV, breakdown viscosity; FV, final viscosity; SV, setback viscosity; PT, pasting temperature.

In order to compare the relationships between the starch properties of highland barley varieties of different colors, hierarchical clustering was performed by granule size, apparent amylose, molecular weight, chain length distribution parameters, relative crystallinity, 1045/1022 and 1022/995, water solubility and swelling power (95 °C), and thermal and pasting properties. The dendrogram created in this study consisted of two main clusters, as shown in Figure 7. According to the differences between the starch parameters, one cluster contained only one highland barley variety (YHB) and the other three varieties were included in other groups, and the relative crystallinity, 1045/1022 cm^−1^, 1022/995 cm^−1^, molecular weight parameters, and pasting viscosity values of YHB were higher. The remaining varieties were further divided into two groups. One group contained blue highland barley (BLHB), and the other contained black highland barley (BHB) and purple highland barley (PHB), among which the AM, PDI, and AP_L_ values were higher in BHB and PHB. The results show that although the yellow highland barley starch was significantly different from the other highland barleys, the physicochemical properties of starch in highland barley of different colors are different.

## 4. Conclusions

In this study, the starch characteristics of black, purple, blue, and yellow highland barley varieties were investigated. All highland barley starches had smooth oval and disc shapes, and their crystallinities varied between 25.67% (PHB) and 27.59% (YHB). The granule size and apparent amylose content of the different highland barley starches varied, but all showed a typical A-type crystalline structure. In addition, the relative crystallinity, 1045/1022, Mw, and RSL of YHB were higher, which enhanced its intermolecular interactions and formed a stable particle structure, thereby increasing the Tp and PV of the starch. Cluster heat map analysis showed that the fine structure, water solubility, swelling power, thermal parameters, and pasting properties were different between the colored highland barley starches and were influenced by the genotype of the varieties. Overall, the selection of colored highland barley varieties with different starch characteristics is critical for meeting diverse food product needs, providing a reference for the high-quality breeding and utilization of highland barley varieties of different colors.

## Figures and Tables

**Table 1:** Granule size distribution parameters ^2^ and apparent amylose of highland barley starch from four cultivars ^1^.

Samples	Granule Size Distribution (μm)			Apparent Amylose (%)
	d (0.5)	D [3, 2]	D [4,3]	
BHB	16.96 ± 0.04 ^c^	9.23 ± 0.01 ^c^	16.93 ± 0.05 ^c^	19.71 ± 0.64 ^ab^
PHB	16.63 ± 0.05 ^d^	9.05 ± 0.02 ^d^	16.64 ± 0.06 ^d^	20.26 ± 0.43 ^a^
BLHB	18.65 ± 0.03 ^a^	9.89 ± 0.01 ^a^	18.66 ± 0.04 ^a^	18.58 ± 0.40 ^c^
YHB	17.27 ± 0.06 ^b^	9.34 ± 0.02 ^b^	17.30 ± 0.07 ^b^	18.88 ± 0.24 ^bc^

^1^ The data are expressed as the mean ± standard deviation, *n* = 3. Different letters in the same column indicate significant differences, *p* < 0.05. ^2^ d (0.5) is the diameter corresponding to 50% of the cumulative distribution of particle size. D [3, 2] and D [4,3] are the surface-weighted and volume-weighted average diameters of the particles, respectively. Note: BHB, PHB, BLHB, and YHB represent black highland barley, purple highland barley, blue highland barley, and yellow highland barley, respectively.

**Table 2:** Molecular weight and chain length distribution parameters, relative crystallinity, and IR ratio of highland barley starch from four cultivars ^1^.

Samples	Molecular Weight Distribution					Chain Length Distribution				Relative Crystallinity (%)	1045/1022 (cm^−1^)	1022/995 (cm^−1^)
	Mw (×10^7^ g/mol)	Mn (×10^7^ g/mol)	Mp (×10^7^ g/mol)	PDI	Rz (nm)	APs (%)	AP_L_ (%)	AM (%)	RSL			
BHB	5.47 ± 0.00 ^c^	1.99 ± 0.00 ^d^	11.13 ± 0.00 ^c^	2.74 ± 0.00 ^a^	132.8 ± 0.3 ^d^	56.35 ± 0.10 ^c^	21.99 ± 0.14 ^a^	21.65 ± 0.12 ^b^	2.56 ± 0.01 ^c^	26.56 ± 0.52 ^b^	0.700 ± 0.00 ^b^	0.812 ± 0.01 ^b^
PHB	5.83 ± 0.01 ^b^	2.14 ± 0.00 ^b^	11.20 ± 0.01 ^b^	2.72 ± 0.00 ^b^	141.2 ± 0.5 ^b^	55.95 ± 0.16 ^d^	21.94 ± 0.07 ^a^	22.18 ± 0.10 ^a^	2.55 ± 0.02 ^c^	25.67 ± 0.31 ^c^	0.701 ± 0.00 ^b^	0.812 ± 0.01 ^b^
BLHB	5.43 ± 0.00 ^d^	2.07 ± 0.00 ^c^	10.78 ± 0.00 ^d^	2.62 ± 0.00 ^c^	134.8 ± 0.6 ^c^	58.17 ± 0.17 ^b^	21.24 ± 0.10 ^b^	20.59 ± 0.13 ^c^	2.74 ± 0.01 ^b^	27.12 ± 0.23 ^ab^	0.703 ± 0.00 ^b^	0.803 ± 0.00 ^b^
YHB	6.72 ± 0.01 ^a^	2.68 ± 0.01 ^a^	11.54 ± 0.00 ^a^	2.53 ± 0.01 ^d^	142.4 ± 0.2 ^a^	61.62 ± 0.27 ^a^	21.36 ± 0.10 ^b^	16.93 ± 0.22 ^d^	2.88 ± 0.00 ^a^	27.59 ± 0.42 ^a^	0.730 ± 0.01 ^a^	0.834 ± 0.00 ^a^

^1^ Data are expressed as mean ± standard deviation, *n* = 3. Different letters in same column indicate significant differences, *p* < 0.05. Note: BHB, PHB, BLHB, and YHB represent black highland barley, purple highland barley, blue highland barley, and yellow highland barley, respectively.

## Data Availability

The original contributions presented in the study are included in the article/Supplementary Materials, further inquiries can be directed to the corresponding author.

## Supplementary Materials

The following supporting information can be downloaded at: https://www.mdpi.com/article/10.3390/foods14020186/s1, Table S1: Chemical components of highland barley starch from four cultivars; Table S2: Water solubility and swelling power of highland barley starch from four cultivars; Table S3: Thermal and pasting properties of highland barley starch from four cultivars; Figure S1: Bivariate flow cytometric histograms of highland barley starch from four cultivars; Figure S2: Color characteristics of highland barley grain from four cultivars (A); Correlation analysis between AAC and color characteristics (B).
