# Supplementary material for: Characterization and Comparison of Structure and Physicochemical Properties of Highland Barley Starch of Different Colors
Source: Foods. 2025 Jan 9;14(2):186. doi: 10.3390/foods14020186 (PMC11765291; doi:10.3390/foods14020186)
Supplement: Supplementary file 1 [file foods-14-00186-s001.zip › foods-3362665-supplementary.pdf]

Table S1

Chemical components of highland barley starch from four cultivars [a].

Samples	Total starch (%)	Protein (%)	Crude fiber (%)	Crude fat (mg/g)	Moisture content (%)
BHB	64.39±2.48 ^{a}	15.74±0.33 ^{a}	2.48±0.06 ^{a}	10.98±0.70 ^{a}	10.90±0.36 ^{a}
PHB	64.02±2.35 ^{a}	14.46±0.24 ^{ab}	2.40±0.09 ^{a}	10.47±1.39 ^{a}	8.10±0.08 ^{b}
BLHB	62.10±3.42 ^{a}	14.03±1.58 ^{ab}	1.11±0.03 ^{c}	5.48±0.50 ^{b}	7.56±0.18 ^{c}
YHB	61.70±1.13 ^{a}	10.72±3.18 ^{b}	1.39±0.02 ^{b}	9.72±1.77 ^{a}	6.38±0.12 ^{d}

[a] Data are expressed by mean ± standard deviation, n = 3. Different letters in the same column indicate significant differences, $p < 0.05$.

Note: BHB, PHB, BLHB, and YHB represent black highland barley, purple highland barley, blue highland barley, and yellow highland barley, respectively.

Table S2

Water solubility and swelling power of highland barley starch from four cultivars [a].

Samples	Water solubility (%)			Swelling power (g/g)		
	75℃	85℃	95℃	75℃	85℃	95℃
BHB	4.19±0.28 ^{a}	6.02±0.59 ^{b}	10.31±0.20 ^{c}	8.61±0.36 ^{b}	10.50±0.24 ^{b}	13.17±0.95 ^{bc}
PHB	4.52±0.11 ^{a}	7.59±0.57 ^{a}	17.89±0.73 ^{a}	8.57±0.27 ^{b}	10.54±0.26 ^{a}	14.51±0.37 ^{b}
BLHB	3.25±0.18 ^{b}	5.97±0.35 ^{b}	12.97±0.38 ^{b}	7.39±0.44 ^{c}	9.52±0.47 ^{b}	12.31±0.95 ^{c}
YHB	3.36±0.06 ^{b}	6.19±0.46 ^{b}	13.08±0.79 ^{b}	11.21±0.74 ^{a}	13.09±0.68 ^{b}	17.95±0.34 ^{a}

[a] Data are expressed by mean ± standard deviation, n = 3. Different letters in the same column indicate significant differences, $p < 0.05$.

Note: BHB, PHB, BLHB, and YHB represent black highland barley, purple highland barley, blue highland barley, and yellow highland barley, respectively.

Table S3

Thermal and pasting properties of highland barley starch from four cultivars [a].

Samples	Thermal parameters [b]		Pasting parameters [c]					
	Tp (°C)	ΔH (J/g)	PV (cP)	TV (cP)	BV (cP)	FV (cP)	SV (cP)	PT (°C)
BHB	59.56±0.04 [b]	9.43±0.31 [b]	2416±15 [b]	1920±8 [b]	496±7 [b]	2596±23 [b]	676±15 [b]	90.56±0.04 [b]
PHB	59.33±0.17 [b]	9.79±0.28 [ab]	1713±46 [c]	1445±28 [c]	268±19 [c]	1936±46 [c]	497±34 [c]	89.33±0.42 [c]
BLHB	60.16±0.08 [a]	9.94±0.52 [ab]	1642±14 [d]	1448±2 [c]	194±13 [d]	1716±17 [d]	268±18 [d]	93.76±0.02 [a]
YHB	60.19±0.25 [a]	10.78±0.89 [a]	2660±27 [a]	2012±14 [a]	648±13 [a]	3562±34 [a]	1550±48 [a]	82.38±0.03 [d]

[a] Data are expressed by mean ± standard deviation, $n = 3$. Different letters in the same column indicate significant differences, $p < 0.05$.

[b] Tp, gelatinization peak temperature; ΔH, gelatinization enthalpy.

[c] PV, Peak viscosity; TV, Trough viscosity; BV, Breakdown viscosity; FV, Final viscosity; SV, Setback viscosity; PT, Pasting temperature.

Note: BHB, PHB, BLHB, and YHB represent black highland barley, purple highland barley, blue highland barley, and yellow highland barley, respectively.

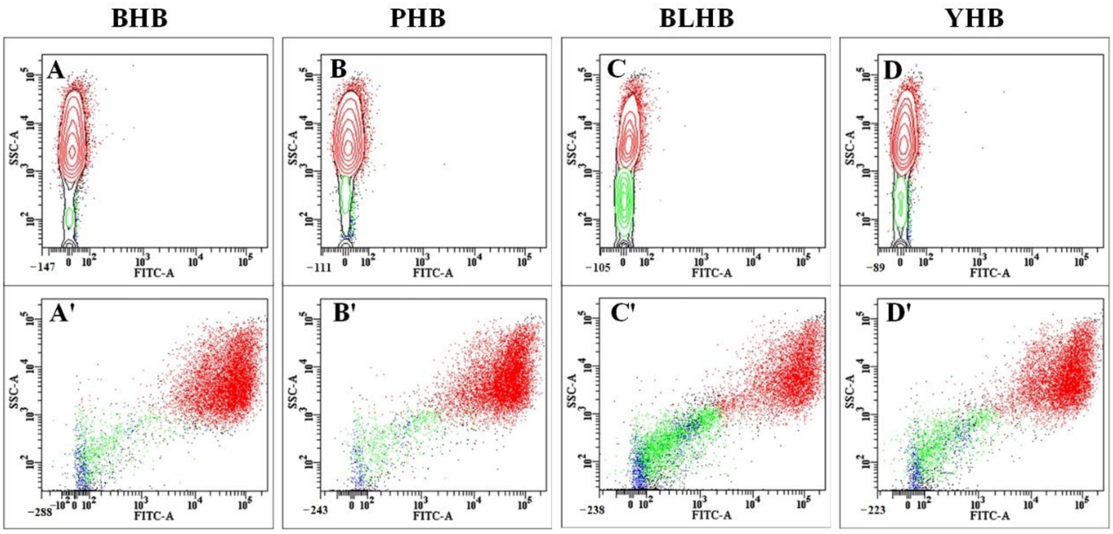

Figure S1. Bivariate flow cytometric histograms of highland barley starch from four cultivars. (**A**–**D**) imaging figure of unstained starch (negative control); (**A'**–**D'**) imaging figure of 1-aminopyrene-3,6,8-trisulfonic acid (APTS) stained starch. Note: BHB, PHB, BLHB, and YHB represent black highland barley, purple highland barley, blue highland barley, and yellow highland barley, respectively.

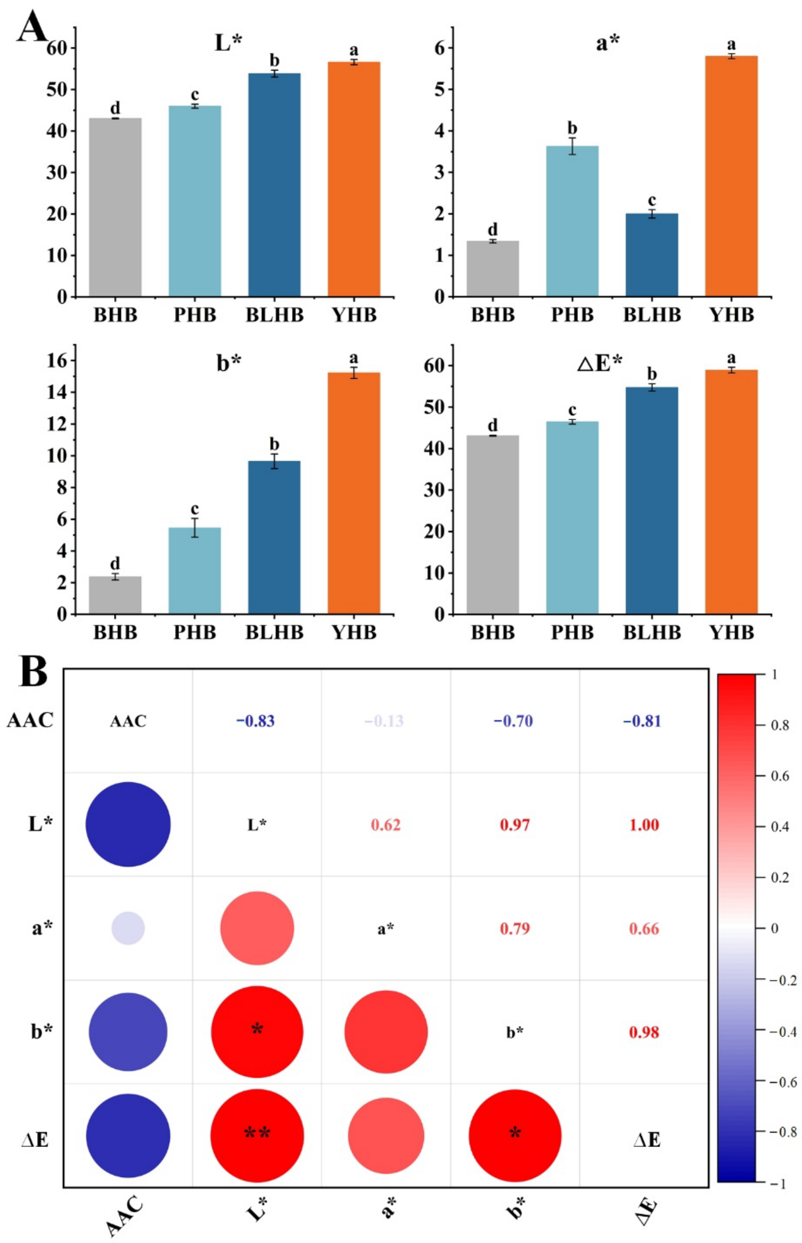

Figure S2. Color characteristics of highland barley grain from four cultivars (A); Correlation analysis between AAC and color characteristics (B). Different letters indicate significant differences, $p < 0.05$. Note: BHB, PHB, BLHB, and YHB represent black highland barley, purple highland barley, blue highland barley, and yellow highland barley, respectively. * $p \leqslant 0.05$, ** $p \leqslant 0.01$.

Supplementary Methods

Color measurements

The color parameters of highland barley grains were measured by colorimetry using Colorimeter Ci7600 (Aisaili Color Technology Inc., Shanghai, China). Standard whiteboards were selected for calibration (Ls: 100, as: 0, bs: 0), and the color was expressed by L* (lightness), a* (+a*: red, -a*: green), and b* (+b*: yellow, -b*: blue). The color intensity (ΔE) was calculated as follows:

ΔE*=$\sqrt{(L_s - L^*)^2 + (a_s - a^*)^2 + (b_s - b^*)^2}$.
